# Feasibility Analysis of Interleukin-13 as a Target for a Therapeutic Vaccine

**DOI:** 10.3390/vaccines7010020

**Published:** 2019-02-12

**Authors:** John Foerster, Aleksandra Molęda

**Affiliations:** Department of Molecular and Clinical Medicine, Jacqui Woods Centre, Ninewells Hospital, Medical School, University of Dundee, Ninewells Drive, Dundee DD1 9SY, UK

**Keywords:** vaccine, VLP, IL-13, drug safety, IL-4, atopic dermatitis, asthma

## Abstract

Background: The development of therapeutic vaccines requires thorough knowledge of potential hazards associated with long-term inactivation of self-proteins. Among potential targets, interleukin 13 (IL-13) merits consideration, as monoclonal antibodies disrupting IL-13 signaling are proving to be exceedingly effective in common conditions such as atopic dermatitis. Objective: Given the mass publication of scientific data, an appraisal of safety aspects is challenging. Methods: We here provide a three-fold approach to survey clinically relevant information on off-target effects, both adverse and beneficial, that may potentially be encountered in patients undergoing long-term IL-13 inactivation. First, we review non-clinical data in vivo and in vitro. Second, we summarize safety data accumulating from patients dosed with anti-IL-13 drugs. Third, we exploit human mutation data as well as emerging large-scale genetic datasets (global exome data from 60,000 patients) to obtain information on any association of IL-13-inactivating genetic variants with disease states. In addition, we: (1) dissect the precise efficacy signals obtained with various drugs targeting IL-13 and/or IL-4, and (2) summarize unintended, but potentially beneficial effects of prolonged IL-13 inactivation on several functional systems. Results: Prolonged repression of IL-13 in several thousand patients so far has not uncovered any non-redundant functions of IL-13 in immune defense. Furthermore, missense mutations in the key genes IL-13, IL-13Rα1, IL-13Rα2, IL-4, IL-4Rα are common, while no case reports have been published on any immune deficiency or increased risk of neoplastic disease associated with such mutations, suggesting that these genes do not harbor non-redundant roles in adult outbred humans. In terms of efficacy, data from clinically used drugs strongly suggest that targeting IL-13 only, as opposed to IL-13 and IL-4, may be effective in eczema while being more selective. Importantly, several lines of evidence suggest that inhibition of IL-13 may in fact harbor potentially beneficial effects on non-targeted systems, including glucose metabolism, hepatic fibrosis, and atherosclerosis, suggesting that respective outcomes should be systematically captured in patients dosed with IL-13 interfering drugs. Collectively, available evidence suggests that IL-13 may fulfill safety requirements required for the target of a therapeutic vaccine.

## 1. Introduction

The selective inhibition of cytokine function via monoclonal antibodies has revolutionized the treatment of chronic inflammatory conditions. Despite their phenomenal success, there are some limitations to these drugs, including excessive cost for health care providers, emergence of anti-drug antibodies, and the need for relatively frequent dosing, requiring logistics for shipment and storage of sensitive biological products. Therapeutic vaccines eliciting an endogenous antibody response against cytokines provide a potential solution to overcome these shortcomings.

Aside from the question of efficacy, however, the main concern in the use of vaccines, as well as biologic drugs, is the safety of long-term elimination of self-proteins. Thus, blocking of TNF-α may predispose toward opportunistic infections (recently reviewed in [1]). Similarly, elimination of IL-6 requires prior immunization against hepatitis B to avoid infection [2].

The safety of long-term elimination of a given protein is determined by its non-redundant roles. In order to assess these in a systematic fashion for IL-13, the present review will consider mutually complementary data. First, non-clinical data generated in vitro and in animal models will be reviewed. Obviously, an in-depth review of all IL-13-related data is not intended here. Rather, the focus will be on any toxicity/safety-related findings. The importance of non-clinical data is two-fold. On the one hand, they allow prediction of expected safety hazards in clinical settings, thereby informing monitoring. On the other hand, if the clinical data, as is often the case, does not bear out limiting adverse effects that could have been expected based on non-clinical data, this supports the notion that for a given postulated biological function, the target in question (here IL-13) is indeed redundant. 

The second, and in many ways most informative, data set consists of safety data accumulating in the clinical use of monoclonal antibodies. In this context, three IL-13-targeting drugs are being developed in addition to the IL-4/IL-13 dual-specific drug dupilumab which has already been approved.

A third data set consists of genetic data. These not only include the traditional reporting of human pedigrees or sporadic mutants associated with clinical phenotypes, but also screens for over-representation of IL-13 blocking mutations in infectious diseases, as well as global databases cataloguing frequent mutations that have failed to emerge as associated with clinical phenotypes. If inactivating mutations are found to be of high frequency, then their non-association with disease states represents an informative negative finding.

Finally, this review will summarize data suggesting that blocking IL-13 may harbor unintended but potentially beneficial effects based on non-clinical data. The purpose here is to highlight that systematic and targeted monitoring of such data in patients undergoing IL-13 blocking antibody treatment is essential and required to gauge the clinical relevance of these data. 

## 2. Non-Clinical Data on IL-13 and Susceptibility to Disease

The present review section is based on a PubMed search using the strategy: (IL-13/ IL-13R/ Interleukin-13/ Interleukin-13 receptor AND ‘infection’) in: abstract/title; cut-off 10 October 2018. By way of limitation, this review is not intended to be exhaustive but rather attempts to focus on data bearing a predictive value on disease susceptibility in humans.

### 2.1. Schistosoma and Helminth Susceptibility

A wealth of literature is available examining the possibility of increased susceptibility to *Schistosoma* infection. One group of clinically relevant data are studies of associations of human polymorphisms with disease. Thus, an *IL-13* promoter variant (−1111T/C) was found to confer slightly increased susceptibly to *Schistosoma* infection (T/T genotype protective); however, effects of age, gender, and village (the latter likely reflecting variable genetic background) were in fact found to be many orders of magnitude higher [3]. A study on a Brazilian population found a suggestive protective association of the same promoter allele as well as a non-conserved functional allele (rs20541 → R130Q) against *Schistosoma mansoni* [4]. A French study found association of the same IL-13 variant (rs1800925) and *Schistosoma* infection [5]. Another study did not find association of *Schistosoma* infection and IL-13 but only IL-5 polymorphisms [6]. Studies with genetically altered inbred mice indicate that survival in *Schistosoma* infection is not impaired in IL-13 deficient mice [7] and that granuloma formation is only impaired in combined IL-4/IL-13 ablation [8]. In fact, ablation of IL-13 ameliorates lung fibrosis in this setting (see below on this aspect). A recent review of intestinal helminth infection also showed redundancy between IL-4 and IL-13, indicating that IL-13 deficiency can be compensated for by IL-4 in murine models of infection [9]. Taken together, despite the available data suggesting a role for IL-13 in the containment of infections such as intestinal nematodes [10], no evidence has been found by genetic association or patients that deficiency in IL-13 affects susceptibility or disease severity or natural course in humans in *Schistosoma* infection. Nonetheless, mouse data on inbred strains do suggest that an absence of IL-13 might negatively impact the defense against intestinal parasites [11], suggesting that caution may nevertheless be prudent.

### 2.2. Leishmaniasis

Cutaneous Leishmaniasis (CL) is the most common type of *Leishmania* infection causing large ulcers on exposed parts of the body. IL-13 is believed to be responsible for non-healing progressing cutaneous disease, previously demonstrated on wild type, IL-13^−/−^, IL-4Rα^−/−^ and IL-4^−/−^ mice that encountered *Leishmania major* and *Leishmania mexicana* [12,13,14]. IL-4Rα^−/−^ mice were fully resistant to *L. mexicana*, however IL-4^−/−^ mice developed persistent lesions following disease progression [13]. IL-13^−/−^ and IL-4Rα^−/−^ groups developed lesions similar to wild type upon initial infection. However, those cleared, and the mice recovered, indicating that IL-4 is the prevailing cytokine responsible for further susceptibility. The resistant phenotype was highly improved in double knockout mice for IL-13 and IL-4 in *L. major* infection [14]. A possible explanation for why IL-13^−/−^ mice became resistant to the parasites is that IL-13 is suppressing the functions of interferon-gamma (IFN-γ) and IL-12, which play key roles in clearance as shown in previous investigations, indicating susceptible phenotypes upon their modulation [15,16,17,18]. Thus, an IL-13 vaccine could supress parasite progression to a chronic non-healing state and enhance T helper 1 (Th1) responses, allowing recovery. Certainly, no data exist to suggest that an IL-13 vaccine would exacerbate susceptibility to *Leishmania*.

### 2.3. Viral Infections

Regarding a potential effect of blocking IL-13 on viral infections, available evidence from clinical studies with IL-13 blocking antibodies has so far failed to provide any data suggesting clinically relevant impact. Non-clinical studies have shown that Th2 cytokines, including IL-13, may in fact impair immunity to rhinoviruses or respiratory syncytial virus (RSV) either directly [19,20,21] or indirectly via signal transducer and activator of transcription 6 (STAT6) activation [22] and a recent review [19] summarizes evidence suggesting that blocking IL-33/IL-13 may be beneficial in the treatment of virus-associated lung disease. These viral infections in childhood can leave individuals vulnerable to development of chronic inflammatory diseases in adult life and result in impaired antiviral responses [23,24,25]. For instance, a study of influenza A virus shows that transcriptionally active remnants of the virus can remain in lung cells once infection has cleared and can also contribute to chronic lung disease partially accounted for by IL-13 expression [25]. This susceptibility was proposed to be a cause of increased IL-13 signaling via STAT6 [25]. Severe influenza infection can be observed in people suffering from asthma, which leads to further chronic disease exacerbations post viral infection [26]. Furthermore, there is a link between rhinovirus infection, elevated IL-13 levels and contributions of asthma [27]. Asthmatic children had reduced IFN-β and IFN-λ production in comparison to healthy subjects during rhinovirus (RS-16) encounter, leading to disease exacerbations [28]. Similarly, another study of RS-16 illustrated that IL-4 and IL-13 impaired production of IFN-β and IFN-λ and inhibited toll-like receptor 3 (TLR3) and interferon regulatory transcription factor 3 (IRF3), thus allowing the virus to replicate [23]. It has also been suggested that IL-13 is involved in mucus metaplasia that can increase susceptibility to rhinovirus [29] and decreasing IL-13 levels can reduce mucus production, airway hyperresponsiveness and partly reduce airway eosinophilia during rhinoviral infection [30]. Contribution to asthma exacerbations are also the case for RSV as IL-13 is believed to be a mediator of viral pathogenesis driving pulmonary eosinophilia via the IL-33/ST2 pathway [19,31,32]. IL-13-expressing natural killer cells are found to be a source for airway inflammation [33] and were significantly increased during RSV infection [32]. Furthermore, there is a genetic association between a haplotype at the IL-13-IL-4 gene locus and increased IL-13 production with a correlation in elevated risk of RSV bronchiolitis in childhood [34]. RSV can therefore manifest and significantly worsen asthmatic exacerbations by driving airway inflammation and tissue damage during infection [31]. IL-13 overexpression may also be a susceptibility factor for viral respiratory infection, especially in those suffering from an ongoing chronic inflammatory disease. Collectively, these studies suggest that Th2 cytokines can have a negative impact on antiviral responses and can contribute to future health complications. Taken together, therefore, available data suggest that therapeutic targeting of IL-13 can relieve chronic inflammatory disease and possibly provide better protection during respiratory infections. 

### 2.4. Other Infections

High levels of IL-13 (> 10 pg/mL) in respiratory secretions were found to predispose to life-threatening rhinovirus infection in children younger than two years in a large national survey (*n* = 56,560) from Argentina, suggesting that blocking IL-13 in this setting may in fact be beneficial [27]. Despite a targeted search, a study from China found associations between IL-4 but not IL-13 polymorphisms and coal miners’ pneumoconiosis [35]. *Chlamydia* commonly cause infections of the respiratory and genital tract. IL-13 increases susceptibility to infection as well as severity by driving increased inflammation and bacterial manifestation [36]. Conversely, respiratory *Chlamydia* infection in early life was shown to reduce IL-13Rα2 expression and attenuate IL-13 production with potential secondary benefit later in life on asthma susceptibility [37]. 

### 2.5. Septic Shock

A Spanish study on 48 children with sepsis found significantly lower IL-13 levels in patients six hours after onset of refractory shock [38], while a British study in 31 adults observed a correlation of TNF-α and IL-13 levels and a high level of each with septic shock [39]. As of the cut-off date of this review, there is no PubMed available evidence indicating that IL-13 deficiency has serious effects on specific susceptibility to infectious agents and/or septic shock.

### 2.6. Malignancies

While in vitro studies on IL-13 in various cancer model systems are abundant, no clinical or in vivo mouse data link IL-13 directly to pro-tumor or anti-tumor effects to date. Noteworthy are studies linking IL-13 to cancer-associated fibrosis in Hodgkin’s lymphoma and pancreatic cancer [40,41,42,43], tying in with a general pro-fibrotic effect of IL-13 (discussed below). Also, the abundance of IL-13Rα2 on glioblastoma cells was clinically explored by designing an IL-13-linked toxin to kill tumor cells in a phase I clinical trial [44]. There are data on IL-13 promoter polymorphisms as well as IL-13 serum levels in a variety of cancer entities of various stages, but no clear causality patterns have emerged to date. Emerging data from IL-13 blocking clinical trials so far confirm the absence of notable effects on cancer development (see below). However, confidence in the safety of vaccine-based IL-13 blocking will necessarily be increased upon the absence of safety signals in widespread and prolonged clinical dosing with anti-IL-13 drugs in patients.

## 3. Safety Signals from Clinical Trials and Post-Marketing Surveillance

**Figure 1:**
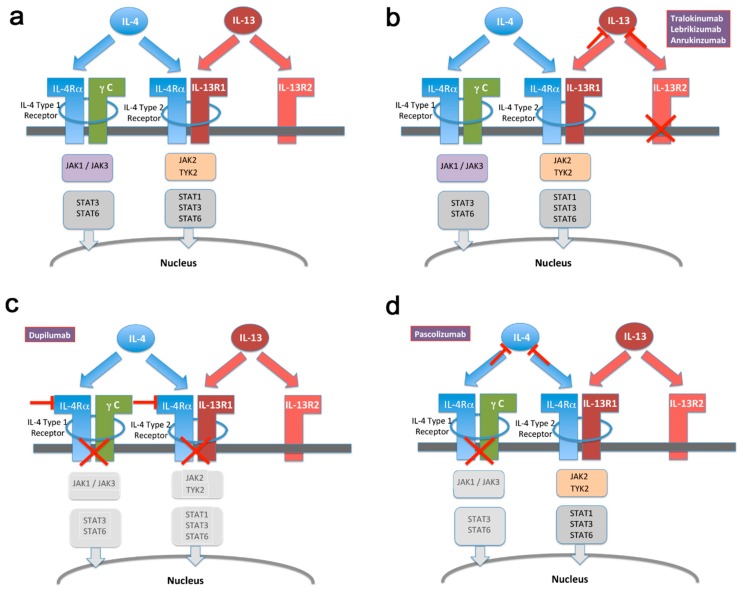
Schematic of IL-4 and IL-13 mediated signal transduction. (**a**) Illustration of overlapping as well as non-redundant signaling mechanisms employed by IL-4 and IL-13, respectively. For details, see text. (**b**–**d**) Same as in (**a**) with highlighted drugs targeting specific molecules, respectively. Drug-mediated inactivation of pathway components is illustrated by grey-shading of respective boxes. STAT = signal transducer and activator of transcription. JAK = Janus kinase. TYK = tyrosine kinase.

Dupilumab, a dual-acting drug blocking both IL-4 and IL-13 function (Figure 1b) was approved by the Food and Drug Administration (FDA) and European Medicines Agency (EMA) in 2017 for use in atopic dermatitis. Approval for use in asthma is expected for Q4 2018. Recent phase III [45] (*n* = 210), [46] (*n* = 1908) studies on asthma showed no significant and/or severe treatment emergent infections. The same was previously found in published studies on atopic dermatitis [47] (*n* = 325), [48] (*n* = 1379). No long-term safety concerns have emerged either as part of the Regeneron Open Label Extension (OLE) study, featuring continuous dosing for a total of three years (*n* = 2000, latest update 2017, REGN668/SAR231893, [49]), nor through post-marketing safety monitoring eighteen months after market introduction in the US. These data are reassuring in terms of safety and are reflected in a recent meta-analysis that identifies conjunctivitis as the only adverse effect emerging from present data [50]. It is worth remembering, however, that patients with a history of helminth infection were excluded from clinical trials.

Currently available safety data for the IL-13 selective monoclonal antibodies confirm the dupilumab data. Thus, for tralokinumab, collectively sizeable phase II populations are available including [51] (*n* = 79), [52] (*n* = 409), [53] (*n* = 452), [54] (*n* = 194), as well as for lebrikizumab, including [55] (*n* = 1081), [56] (*n* = 313), and [57] (*n* = 209). A further anti-IL-13 monoclonal, anrukinzumab, studied in active ulcerative colitis (*n* = 84) also found no significant adverse effects [58]. This study also measured and detected feedback elevation of IL-13 in peripheral blood of treated patients. Overall, available clinical data from patients dosed with anti-IL-4/IL-13 drugs have not uncovered safety signals to date that would suggest a vaccine approach to be unsafe, as reflected in a recent independent meta-review of these data [59]. 

## 4. Evidence for IL13 Related Phenotypes from Large-Scale Genetic Databases

The advent of high-throughput genome wide sequencing has provided several databases compiling phenotypes linked to genetic polymorphisms and/or mutations. In the case of IL-13, a search in the Single Nucleotide Polymorphism Database (dbSNP) for IL-13, IL-13Rα1 and IL-13Rα2 reveals no clinically-relevant phenotype-associated variants. There are data linking IL-13 polymorphisms with the outcome of children with minimal change nephrotic syndrome [60,61], although the significance of this remains uncertain. Furthermore, there are no published pedigrees and / or single patient case reports involving variants mapping to the above listed genes, nor entries listed on Online Mendelian Inhertience in Man (OMIM).

The completion of the Exome Aggregation Consortium (ExAC) sequencing of 60,000 genomes from a variety of global populations has enabled a quantitative assessment of the impact of genetic variants on overall fitness in terms of evolutionary selection. We extracted these data for key genes related to IL-13 signaling. As shown in Table 1, non-conserved and even loss-of-function mutations are frequent. Except for IL-13Rα1 (numbers set in bold), none of the genes exhibit apparent evolutionary selection against homozygous occurrence of loss-of-function mutations. Although the high z-score could suggest that IL-13Rα1 may be important in pre-natal development, IL-13Rα1-deficient mice in fact exhibit normal fertility rates and display no global development abnormality but only a specific defect in M2-type macrophage development [62]. Furthermore, the Exome Variant Server (EVS) compiles allele frequencies for approximately 6000 genomes from European and African ancestries, respectively. We extracted EVS-listed variants for IL-13 relevant genes with high probability of deleterious function based on: (1) non-conserved amino-acid replacement, (2) high probability (score > 0.8) in PolyPhem2-based prediction, and (3) 100% conservation of the main allele between humans and chimpanzee (Table 2). These data show that deleterious variants in these genes are, in fact, surprisingly common, in some cases amounting to 25% of all minor variants identified (Table 2, printed in bold). Therefore, the absence of clinical case studies reporting health issues in individuals harboring any of these mutations in the public space is significant, suggesting that deficient IL-13 signaling caused by congenital mutations is not associated with severe immunodeficiency or failure to thrive. 

## 5. IL-13 on Its Own Represents a More Selective Target Than IL-4/IL-13 in Combination for Treatment of Atopic Conditions

Several drugs targeting IL-13 are undergoing clinical development or already marketed. The precise efficacy, as well as adverse effect profile of each drug is related to its molecular mode of action. As shown in Figure 1a, IL-13 and IL-4 signal through partially overlapping pathways. Both cytokines will activate the so-called type-2 IL-4 receptor, followed by JAK2/Tyk2 phosphorylation and subsequent STAT translocation. By contrast, only IL-4 activates the type-1 IL-4 receptor, whereas IL-13 additionally binds to IL-13α2, commonly regarded as an inhibitory decoy receptor. By binding to IL-4Rα, dupilumab essentially abrogates signaling through both IL-4 and IL-13 (Figure 1b). In contrast, pascolizumab directly binds to IL-4, thereby preserving type-2 receptor activation via IL-13 (Figure 1c). This drug did not show clinical efficacy, thereby suggesting that the effect of this system on asthma and eczema pathogenesis primarily proceeds through the IL-4 type 2 receptor [65]. Finally, IL-13 selective antibodies (Figure 1d) prevent IL-13 binding but preserve the activation of both active receptor systems. The emerging clinical efficacy of these drugs both in asthma and atopic eczema suggests that the more selective inactivation of IL-13 achieves sufficient activity while preserving IL-4 activity, with a potentially important gain in safety profile in terms of immunity to infectious agents [9]. A number of selective as well as global JAK inhibitors are currently also being developed for atopic disease [66], as well as other conditions including cancer. However, the complex nature of precise JAK/STAT coupling of IL-4, as well as other signal transducing pathways [67], prevent an unambiguous mapping of specific adverse effects and/or activity profiles to relative IL-4/IL-13 signaling. 

## 6. Additional Potential Effects of an IL-13 Vaccine

When considering the potential of IL-13 as a vaccine target to improve atopic disease, potential off-target effects in addition to those considered above may occur which, although unintended, may in fact prove of interest for further vaccine applications. Therefore, this final section will summarize data suggesting potential vaccine effects on specific functional systems that warrant dedicated monitoring during clinical applications based on currently available data.

### 6.1. Improvement of Fibrosis

A substantial amount of data indicates that an anti-IL-13 vaccine may be beneficial for the prevention of the amelioration of hepatic fibrosis in *Schistosoma* infection [68] as well as non-infectious fibrosis, as reported in many studies (see above) [69,70,71]. A preventative effect in this regard could indeed constitute a major independent indication for anti-IL-13 vaccination.

### 6.2. Glucose Metabolism

Blocking IL-13 signaling may in fact improve impairment in glucose metabolism [72,73,74] and regulate glucose uptake and pathways involved in skeletal muscle maintenance [75,76]. Conversely, however, IL-13 has also been suggested to fulfill an autocrine anti-inflammatory role in diabetes [77]. 

### 6.3. Atherosclerosis

A somewhat related activity of IL-13, also possibly involving macrophage activation, is regulation of atherosclerosis, where the precise role and mechanism and its clinical significance remain to be elucidated [75]. Animal models of IL-13 deficiency suggest IL-13 may act as a contributor in atherosclerosis [78,79,80]. Other studies suggest that IL-13 leads to atherosclerosis by inducing expression of peroxisome proliferator activated receptor δ (PPARδ) or PPARβ in the adipose tissue and by increasing CD36 levels [81,82]. However, obviously atherosclerosis development is multifactorial and complex. Furthermore, the Th1 and Th2 responses contribute differently during various stages of plaque development, and IL-13 alone cannot reverse disease progression [83,84]. Clearly, a more systematic monitoring of glucose metabolism, as well as markers of systemic inflammation in patients currently dosed with anti-IL-13 drugs, would be important to better understand the clinical significance of the available non-clinical data in this regard.

## 7. Conclusions

The clinical use of dupilumab, as well as selective anti-IL-13 drugs, has shown that blocking IL-13 is effective both for the treatment of asthma and atopic dermatitis and may be of use in other atopic conditions (e.g., severe contact allergy). A precise understanding of the molecular signaling mechanisms suggests that targeting IL-13, while equally effective, preserves IL-4 signaling through the type-2 IL-4 receptor which may constitute an important safety benefit regarding immunity, especially to helminth infections. A large body of genomic data appear to confirm emerging safety data from anti-IL-13/IL-4 clinical use that prolonged blocking of IL-13 is not associated with reduced systemic immunity or opportunistic infections. Notably, it remains to be seen whether the pro-fibrotic activity of IL-13 is of clinically high enough relevance to render a vaccination treatment effective in several conditions involving increased fibrosis (e.g. hepatic fibrosis, pulmonary fibrosis due to various etiologies). Finally, the clinical use of IL-13 drugs should be utilized to specifically monitor the effects of IL-13 blocking on glucose metabolism.

## Figures and Tables

**Table 1:** Global counter-selection against mutations in key genes affecting interleukin 13 (IL-13) signaling. ^1^

Gene	Type of Mutation	Expected (*n*) ^2^	Observed (*n*) ^2^	Metric (z/pLI) ^3^
*IL-13*	Synonymous	25.9	26	−0.01
	Missense	48.4	50	−0.11
	LoF	5.4	3	0.03
*IL-13Rα1*	Synonymous	33.5	25	0.91
	Missense	79.2	80	−0.04
	LoF	**11.6**	**0**	0.98
*IL-13Rα2*	Synonymous	33.3	30	0.35
	Missense	77.8	72	0.32
	LoF	12.0	8	0.00
*IL-4Rα*	Synonymous	138.2	146	−0.41
	Missense	283.0	296	−0.38
	LoF	23.0	7	0.03
*IL-4*	Synonymous	29.7	29	0.08
	Missense	57.3	49	0.54
	LoF	6.0	2	0.17

^1^ Data shown were extracted from the ExAC tool [63] and represent a total of approximately 60,000 genotypes. Half of these are derived from Caucasian samples, the remainder from African/Latino/East Asian, Finnish, South Asian, and Other, respectively (for details, see ExAC website); ^2^ Expected and observed numbers are calculated based on exon number and gene length (for details, see EXAC); ^3^ z scores and “probability of intolerance” (pLI) (for loss-of-function (LoF) express a degree of deviation between expected and observed. The closer the score is to 1, the greater the discrepancy (for details, see EXAC website). Negative scores indicated that observed variants are even more frequent than expected.

**Table 2:** Frequencies of damaging variants in key genes affecting IL-13 signaling. ^1^

Gene	MAF European ^2^	MAF African ^2^	Change	rs ID ^3^
*IL-13*	0.000	0.023	P60L	rs146770163
	0.023	0.000	W68R	rs143660447
	0.047	0.000	L72P	rs148077750
	0.023	0.000	V87G	rs199513811
	0.000	0.023	T96N	rs374668631
	0.000	**0.114**	V125M	rs140196099
*IL-4*	0.000	0.023	R109W	rs373334025
	0.023	0.000	A118V	rs149950065
	0.000	0.023	K150M	rs149147538
*IL-4R*	0.012	0.000	S6C	rs151179009
	0.000	**0.273**	V85M	rs141983833
	0.000	0.023	P169L	rs145473866
	0.000	0.023	R200W	rs370524692
*IL-13Rα1*	0.000	0.026	S153R	rs368784033
	0.000	0.052	N157K	rs138018049
	**0.119**	0.000	R340C	rs139927088
	0.000	**0.261**	H398Y	rs145848479
	0.000	**0.157**	D403N	rs149035999
	0.000	0.026	D414N	rs142037578
*IL-13Rα2*	0.000	0.052	R343C	rs375241228
	0.000	0.026	R248W	rs369836103
	0.000	0.026	T107M	rs368005707
	0.000	0.026	R74Q	rs377163094
	0.000	0.026	P55L	rs370152207

^1^ Data shown were extracted from the Exome Variant Server [64] using the following filters: probability of protein-damaging function >0.8 (PolyPhen2 score); non-conserved protein amino acid replacement; conservation in major allele between Human and Chimpanzee genomes; ^2^ Minor allele frequencies (MAF) for European and African descent populations, respectively where MAF is a percentage among all minor alleles in multi-allelic variants (for details, see EVS website); ^3^ rsID is the dbSNP reference for SNP identifier (for details, see EVS website). Data set in bold print represent MAF > 0.1 in at least one population.
